# Proteasome inhibitors as experimental therapeutics of autoimmune diseases

**DOI:** 10.1186/s13075-015-0529-1

**Published:** 2015-01-28

**Authors:** Sue Ellen Verbrugge, Rik J Scheper, Willem F Lems, Tanja D de Gruijl, Gerrit Jansen

**Affiliations:** Department of Rheumatology, VU University Medical Center, 1081 HV Amsterdam, The Netherlands; Department of Pathology, VU University Medical Center, 1081 HV Amsterdam, The Netherlands; Department of Medical Oncology, VU University Medical Center, 1081 HV Amsterdam, The Netherlands

## Abstract

Current treatment strategies for rheumatoid arthritis (RA) consisting of disease-modifying anti-rheumatic drugs or biological agents are not always effective, hence driving the demand for new experimental therapeutics. The antiproliferative capacity of proteasome inhibitors (PIs) has received considerable attention given the success of their first prototypical representative, bortezomib (BTZ), in the treatment of B cell and plasma cell-related hematological malignancies. Therapeutic application of PIs in an autoimmune disease setting is much less explored, despite a clear rationale of (immuno) proteasome involvement in (auto)antigen presentation, and PIs harboring the capacity to inhibit the activation of nuclear factor-κB and suppress the release of pro-inflammatory cytokines such as tumor necrosis factor alpha and interleukin-6. Here, we review the clinical positioning of (immuno) proteasomes in autoimmune diseases, in particular RA, systemic lupus erythematosus, Sjögren’s syndrome and sclerodema, and elaborate on (pre)clinical data related to the impact of BTZ and next generation PIs on immune effector cells (T cells, B cells, dendritic cells, macrophages, osteoclasts) implicated in their pathophysiology. Finally, factors influencing long-term efficacy of PIs, their current (pre)clinical status and future perspectives as anti-inflammatory and anti-arthritic agents are discussed.

## Introduction

Rheumatoid arthritis (RA) is a common autoimmune disease characterized by synovial inflammation and hyperplasia, autoantibody production, and cartilage and bone destruction, the underlying cause of which lies in immune regulatory factors such as the loss of tolerance [1]. How this process is linked to a localized onset of inflammation in the joint is still unclear but it involves migration and accumulation of immune effector cells, including macrophages and osteoclasts, myeloid and plasmacytoid dendritic cells (DCs), B cells and T cells [1]. Th17 subsets, which produce interleukin (IL)-17 and IL-21, also play a crucial role in the development of RA in combination with limited functional capabilities of regulatory T cells (Tregs). Current therapies for RA rely on early and aggressive treatment with disease-modifying anti-rheumatic drugs (DMARDs), including methotrexate and glucocorticoids and/or biological agents. These mostly include antibodies to pro-inflammatory cytokines, for example, tumor necrosis factor (TNF)α and IL-6 and others, including rituximab (anti-CD20) and abatacept (CTLA4 IgG1 fusion protein), which also interfere with the underlying immune/inflammatory events. In general, monotherapy with DMARDs has limited long-term efficacy, probably as a consequence of multidrug resistance. Large clinical studies with long-term follow-up demonstrated that the use of combinations of conventional DMARDs, particularly methotrexate, with biological agents was highly effective in achieving clinical remission and preventing radiological deterioration in approximately 50% of RA patients, but the remaining 50% of patients still experienced insufficient disease activity reduction or sustained active disease [2]. Therefore, in RA treatment there is still room for investigational new drugs with novel mechanisms of action, including antibody-guided and small molecule-mediated targeting of specific cell types (T/B cells, macrophages, synoviocytes), cytokines and their receptors, and intracellular (signaling) pathways [1]. Janus kinase inhibitors and Spleen kinase inhibitors represent examples of these latter drugs, displaying great pre-clinical potential, but, as with biological agents, safety/toxicity issues apply [3].

Proteasome inhibitors (PIs) may also fall in the category of potentially attractive investigational drugs for their ability to (a) inhibit the activation of nuclear factor (NF)-κB and transcriptional regulation of pro-inflammatory cytokine release, and/or (b) induce apoptosis of activated immune cells. The rationale of PIs to act as anti-inflammatory agents in the treatment of autoimmune diseases, including RA, systemic lupus erythematosus (SLE), Sjögren’s syndrome (SS) and scleroderma, has been the subject of several recent publications and reviews [4-6]. In this review, we cover the diversity and relevance of constitutive and immunoproteasome subtypes in immune competent cells involved in autoimmune diseases, and provide an overview of several classes of reversible and irreversible PIs for therapeutic interventions. Consistent with the chronic nature of the disease, it is also of relevance to expand our knowledge on the efficacy of PIs following long-term PI administration, and the possible acquisition of resistance to PIs. This review also elaborates on this issue.

## Proteasome subtypes

**Figure 1:**
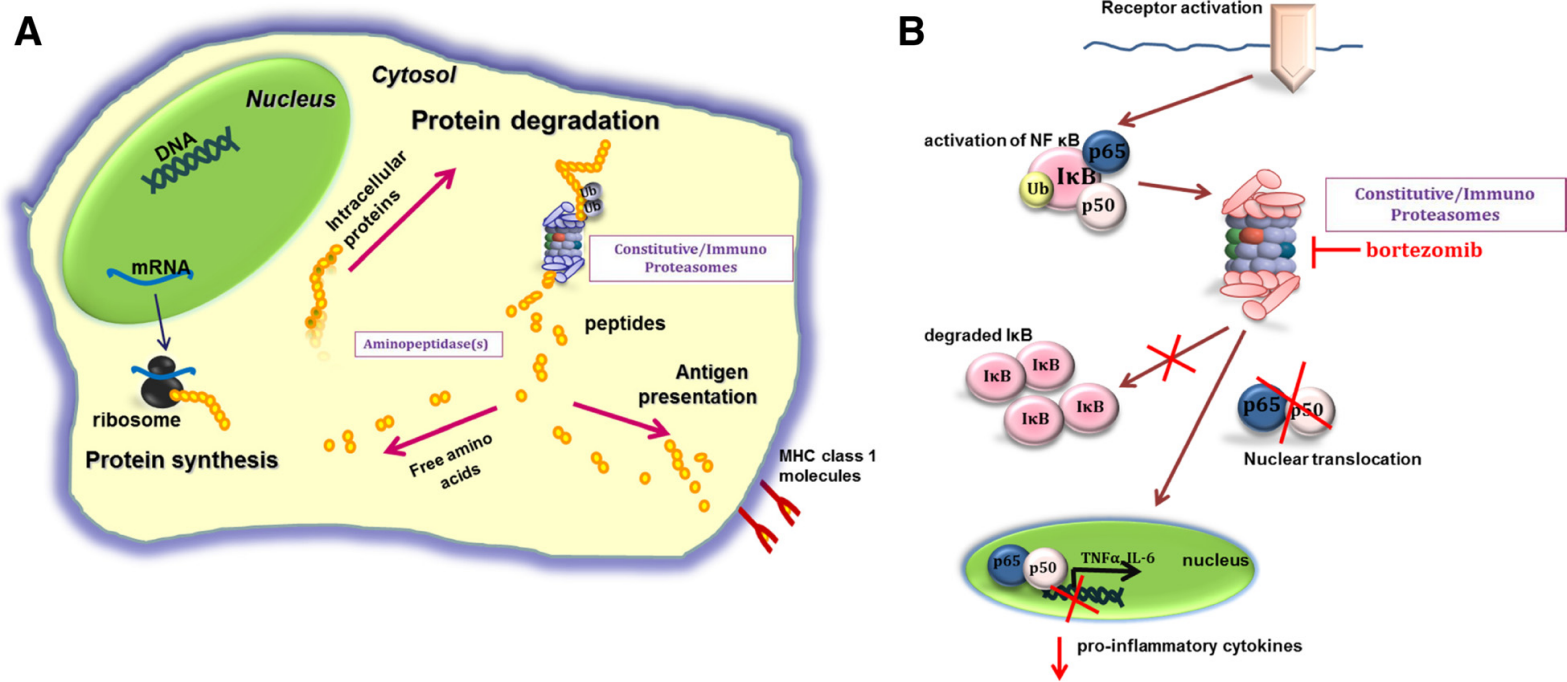
**Role of proteasomes in protein degradation and nuclear factor-κ** **B activation. (A)** After initial synthesis, proteins at the end of their (functional) life-span, or damaged/misfolded proteins, are subject to degradation after conjugating with an ubiquitin (Ub) tag. Recognition by the proteasome initiates protein degradation to smaller peptides, which are further processed by aminopeptidases either to free amino acid for renewed protein synthesis or to trimmed peptides presented by major histocompatibility complex class I molecules. **(B)** Mechanism of blockade of nuclear factor (NF)-κB activation by the proteasome inhibitor bortezomib. This inhibitory effect prevents the degradation of the natural inhibitor of NF-κB (that is, IκB) along with nuclear translocation of p50/p65 and transcription of pro-inflammatory cytokines. IL, interleukin; TNF, tumor necrosis factor.

**Figure 2:**
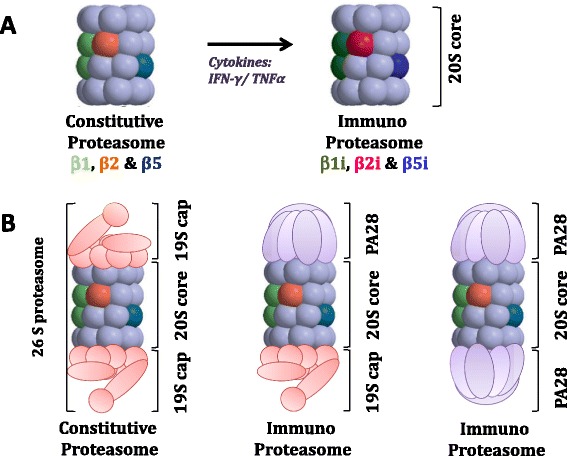
**Subunit composition of constitutive and immunoproteasomes. (A)** 20S core proteasome. **(B)** Fully assembled proteasome. Coloured subunits represent catalytic subunits. IFN, interferon; TNF, tumor necrosis factor.

The ubiquitin-proteasome system (UPS) plays a central role in maintaining cellular homeostasis by controlling the timely breakdown of many key proteins, including those involved in cell cycle regulation, activation of transcription factors (for example, NF-κB) and apoptosis induction (Figure 1A,B). The proteasome has a 26S structure, which consists of the 19S regulator and the 20S central proteolytic core (Figure 2A). Three β-subunits within the 20S core of the proteasome harbor its catalytic activity: the β5 subunit (PSMB5, chymotrypsin-like activity), the β1 subunit (PSMB6, caspase-like activity) and the β2 subunit (PSMB7, trypsin-like activity) [7]. Upon stimulation by pro-inflammatory stimuli, for example interferon (IFN)-γ or TNFα, these constitutive proteasome subunits can be replaced by immunoproteasome subunits β5i (PSMB8, LMP7), β1i (PSMB9, LMP2) and β2i (PSMB10, MECL1) and to assemble immunoproteasomes, along with PA28 as regulatory cap (Figure 2B). Immunoproteasomes are mainly found in cells of hematological origin in which they convey specialized functions, including: a) facilitating endogenous antigen presentation via major histocompatibility complex (MHC) class I; b) splicing of antigenic peptides and cross-presentation of exogenous antigens via MHC class I on DCs; and c) preserving protein homeostasis after IFN-γ-induced oxidative stress [5,8]. Beyond this, hybrid variants of immunoproteasomes with constitutive subunits also have been identified in murine heart tissue as well as in human liver, colon, small intestine, kidneys, tumor cells and DCs [9]. These hybrid forms displayed unique antigen-processing properties, thereby expanding the repertoire of antigen presentation by specific cells. Apart from constitutive proteasomes and immunoproteasomes, a third proteasome variant, designated thymo-proteasomes, was identified in cortical thymic epithelial cells. Their function seems to be required for positive selection of CD8+ T cells and in the control of cytokine release [10].

## Proteasomes in inflammatory/autoimmune diseases

The prominent role of the UPS in multiple cellular processes, including MHC-mediated antigen presentation, cytokine and cell cycle regulation and apoptosis, renders it crucial in the development and progression of inflammatory and autoimmune diseases [5]. Given that cytokines are regulatory factors in the formation of immunoproteasomes, it is conceivable that their increased levels would coincide with chronic inflammation. Indeed, elevated immunoproteasome levels have been associated with inflammation and the development and progression of autoimmunity [11,12]. However, it is still a controversial issue whether or not immunoproteasomes drive inflammatory diseases or merely reflect the consequence of excessive cytokine synthesis or cell stress. From knock-out mice experiments, it was concluded that immunoproteasomes have a protective function against the development of autoimmunity [13]. This is supported by the finding that triple knock-out mice for all three immunoproteasome subunits displayed such a markedly altered repertoire of antigenic peptides for MHC class I presentation that they triggered an immune response to mice splenocytes [14]. Of note, mutations in *PSMB8* (β5i) were implicated in aberrant immunoproteasome assembly and function causing human disorders linked to an auto-inflammatory pathogenesis [15]. As an alternative function, Seifert and colleagues [8] suggested that immunoproteasomes have an increased intrinsic catalytic activity relative to constitutive proteasomes and thereby prevent the accumulation of degradation substrates that would otherwise aggregate during inflammation. Studies by Nathan and colleagues [16], however, reported that constitutive and immunoproteasomes bound and degraded ubiquitin conjugates at similar rates and that immunoproteasomes did not protect against experimental autoimmune encephalomyelitis, even though immunoproteasome activity did increase the generation of peptides for MHC class I presentation.

Interestingly, circulating proteasomes were found in serum samples of patients with autoimmune myositis, SLE, primary SS, RA, and autoimmune hepatitis [17-19]. These circulating 20S proteasomes contained both constitutive and immunoproteasome subunits and might serve as potential biomarkers as each disease displayed different proteasome patterns. Moreover, the increased levels of circulating proteasomes in autoimmune diseases might actually function as autoantigens that could induce an autoimmune response. In fact, anti-proteasome autoantibodies were detected in sera of patients with RA, SLE, myositis and multiple sclerosis [18,20,21]. These antibodies interfere with the interaction between the 20S proteasome and P28 and thereby block proteasome activation and functional capacity.

## First and second generation proteasome inhibitors

**Figure 3:**
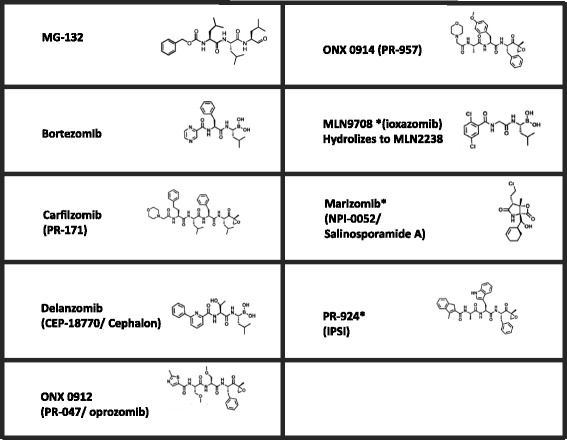
**Chemical structures of proteasome inhibitors.** Asterisks indicate that the compound has not been evaluated for potential anti-inflammatory properties.

The important role of the proteasome in the activation of NF-κB has initiated research to develop PIs for therapeutic interventions for chronic inflammatory diseases and cancer. In a recent historical overview, Goldberg [22] described the timeline of PI development starting more than 40 years ago. Original studies on biochemical mechanisms of protein degradation emerged with the development of MG-132, a peptide aldehyde that blocked proteasome function. About a decade ago, bortezomib (BTZ), a boronic acid peptide, was the first PI that was approved for treatment of therapy-refractory multiple myeloma [23]. Development of BTZ as an anti-inflammatory drug has taken a slower path, but preclinical evaluations are still ongoing [22]. Recent reviews by Huber and Groll [24], and Kisselev and colleagues [7] summarized chemical and crystallography data of BTZ and second-generation PIs designed to target and bind either reversibly or irreversibly to constitutive and/or immunoproteasomes. PIs can be grouped into seven classes: aldehydes, vinyl sulfones, vinylamides (syrbactins), boronates, α’,β’-epoxyketones, α-ketoaldehydes (glyoxals), and β-lactones [24]. Figure 3 and Table 1 provide an overview of the chemical structure and other features (class, target, route of administration) of PIs that are currently under clinical development. All of these PIs represent active site inhibitors, interacting with the amino-terminal Thr1 site of the proteasome catalytic subunits. Apart from these types of PIs, non-competitive PIs have also been developed, which bind to structural non-active subunits (for example, proteasome subunit alpha type, α-subunits) or to regulatory particles outside the proteasome catalytic core [25]. Hereafter, we further elaborate on active-site PIs under pre-clinical evaluation as anti-inflammatory agents.

**Table 1:** **Properties of proteasome inhibitors and clinical administration route**

**Proteasome inhibitor**	**Class**	**Target(s)**	**Administration route**
MG-132	Aldehyde	CP and IP	Not known
Bortezomib	Boronate	CP and IP	Intravenous/subcutaneous
Carfilzomib (PR-171)	α’,β’-Epoxyketone	CP and IP	Intravenous
Delanzomib (CEP-18770/cephalon)	Boronate	CP and IP	Oral
ONX 0912 (PR-047/oprozomib)	α’,β’-Epoxyketone	CP and IP	Oral
ONX 0914 (PR-957)	α’,β’-Epoxyketone	IP	Intravenous
MLN9708* (ioxazomib): hydrolizes to MLN2238	Boronate	CP and IP	Oral
Marizomib* (NPI-0052/salinosporamide A)	β-Lactone	CP and IP	Intravenous and oral
PR-924* (IPSI)	α’,β’-Epoxyketone	IP	Intravenous

CP targeting refers primarily to PSMB5 (β5) subunit; IP targeting refers primarily to PSMB8 (β5i) subunit. Asterisks indicate that the compound has not been evaluated for potential anti-inflammatory properties. CP, constitutive proteasome; IP, immunoproteasome; IPSI, immunoproteasome-specific inhibitor.

## Effects of inhibition of the proteasome system on immune effector cells

Pre-clinical and clinical studies have demonstrated that BTZ elicits immunosuppressive effects either by interfering in the NF-κB signaling pathway or by inducing impaired development or depletion of specific blood cell types [26-29]. It is well recognized that particularly (malignant) plasma cells as professional antibody secreting cells are extremely sensitive to PIs. Mechanistically, proteasome inhibition leads to aberrant degradation of defective ribosomal products in the endoplasmatic reticulum. The overwhelming endoplasmatic reticulum stress provoked by PIs coincides with PI-induced inhibition of NF-κB activation, dampening an anti-apoptotic response. Together, these dual effects lead to the terminal unfolded protein response and apoptotic cell death in myeloma cells as well as in normal plasma cells [30-32]. Table 2 summarizes the impact of BTZ (first generation) and next-generation PIs on various types of immune effector cells (T cells, B cells, DCs, monocytes, macrophages, osteoclasts), which is discussed in greater detail below.

**Table 2:** **Overview of effects of proteasome inhibitors on immune cell function**

	**Macrophages**	**T cells**	**B cells (plasma cells)**	**Dendritic cells**	**Osteoclasts**
MG-132	↓ activation + pro-inflammatory cytokine production	Not documented	Not documented	Not documented	↓ RANKL-induced osteoclast differentiation and function
	↑ anti-inflammatory cytokine production				
Bortezomib	↑ ABCA1 and ABCG1 expression	↑ induction of apoptosis in activated and proliferating cells	↓ plasma cells and autoantibody levels in lupus model	↓ CD40, CD86, CD80, HLA-DR, CD206 and CD209 CD83 expression	↓ osteoclasto-genesis and differentiation
		Depletion of alloreactive T cells and ↓ Th1 cytokines		↑ apoptosis by ↑ bax	↑ osteoclasto-genesis and bone destruction
		↓ release of NF-κB-inducible cytokines by activated T cells from RA patients		↓ TLR and Rel A and B activation	
		↓ activation, proliferation, survival and important immune functions of human CD4+ T cells		↓ T-cell stimulation capacity	
				↓ TLR trafficking, IFN-α and IL-6	
Carfilzomib (PR-171)	Not documented	Not documented	↓ plasma cells lupus model	↓ TLR trafficking, IFN-α and IL-6	Not documented
				↑ apoptosis induction	
Delanzomib (CEP-18770/ cephalon)	Not documented	Not documented	↓ plasma cells and autoantibody levels in lupus model	Not documented	Not documented
ONX0914 (PR-957)	↓ IL-23 release	↓ IFN-γ and IL-2	↓ plasma cells and autoantibody levels in arthritis model	↓ TLR trafficking, IFN-α and IL-6	Not documented
		Shift from IL-17 to Tregs		↑ apoptosis	
				↓ DC differentiation and maturation	

ABCG1/A1, ATP-binding cassette G1/A1; DC, dendritic cell; IFN, interferon; IL, interleukin; NF-kB, nuclear factor kappa beta; RA, rheumatoid arthritis; RANKL, receptor activator of nuclear factor-kB ligand; TLR, Toll-like receptor; Treg, regulatory T cell.

### Monocytes, macrophages and osteoclasts

Macrophage precursors are released into the circulation as monocytes, from where they migrate into tissues. Based on their location and functional phenotype, they are divided into subpopulations harboring both protective and pathogenic functions. Pro-inflammatory cytokines, for example, TNFα, IL-18, IL-12 and IL-23, released from M1-like macrophages have been identified as important mediators in several autoimmune diseases. Consequently, options for therapeutic interventions, including for RA, might include non-specific and specific targeting of M1 macrophages and their products [1]. In this context, Qureshi and colleagues [33] showed that the proteasome has a central role in the regulation of macrophage function by inhibiting the proteasome with several naturally occurring PIs and showing that this suppressed the induction of nitric oxide along with the production of pro-inflammatory cytokines. Also, inhibition of the proteasome with the broad specificity PIs lactacystin or MG-132 suppressed activation of NF-κB in macrophages in conjunction with inhibition of the pro-inflammatory mediators TNF-α and IL-8, while increasing the release of the anti-inflammatory mediator IL-10. More selective inhibition of the immunoproteasome subunit PSMB8 (β5i) by the immunoproteasome inhibitor ONX 0914 (formerly designated PR957) attenuated progression of experimental arthritis by blocking production of IL-23 by activated monocytes [27].

Osteoclasts represent specialized bone-resident macrophages involved in bone remodeling through bone resorption. This process is often affected in chronic inflammatory diseases, leading to accelerated bone loss triggered by the receptor activator of nuclear factor-κB. This receptor, belonging to the TNF superfamily, is expressed on osteoclasts and is a critical stimulator of the differentiation and functional activity of these cells and thus of promotion of bone resorption [1].

The impact of BTZ on human osteoclastogenesis is not fully resolved, with one study reporting beneficial effects on bone resorption [34], and another noting aggravation of bone resorption by BTZ treatment [35]. Whether or not these differential effects are explained by utilization of different experimental models (TNFα-induced versus adjuvant-induced) merits further exploration. It cannot be excluded that effects of BTZ on bone are indirect as osteoclastogenesis is upregulated in active disease; suppression of the inflammatory process by PI treatment would then counteract the elevated bone resorption.

### T cells

Crucial for the function of T lymphocytes are T-cell receptor engagement, co-stimulation and distinct cytokine receptor ligation, which promote their activation and/or differentiation into specialized subsets. Mounting evidence indicates that the inappropriate regulation of Th17 subsets, which produce IL-17 and IL-21, plays a crucial role in the development of a wide range of autoimmune disorders, including RA and SLE [36]. Th17 cells are developmentally closely related to Tregs, whose defective function has been put forward as a cause of tolerance failure in several human autoimmune diseases, including RA, psoriasis and granulomatosis with polyangiitis. Indeed, conversion of immunosuppressive Tregs to pro-inflammatory Th17 cells under the influence of IL-6, IL-23 and transforming growth factor-β, may play an important role in the generation and/or aggravation of autoimmunity [1].

The importance of proteasome catalytic activity in the activation of T cells has been underscored as PIs have been extensively explored for their possible immunosuppressive properties in the treatment of deregulated and unwanted T-cell-mediated immune responses, including those that contribute to the pathogenesis of autoimmune diseases. Direct effects of constitutive and immunoproteasome inhibition by BTZ, epoxomycin and lactacystin on T cells include induction of apoptosis in activated and proliferating cells as well as suppression of activation, proliferation, survival and important immune functions of Th cells [28]. Additionally, Van der Heijden and colleagues [37] showed that BTZ inhibited the release of NF-κB-inducible cytokines by activated T cells from RA patients. Other studies indicated that PSMB8 (β5i) inhibition in T cells can serve as a mechanistic basis for the attenuation of autoimmune diseases. Specifically, Muchamuel and colleagues [27] showed that selective inhibition of the immunoproteasome by ONX 0914 (PR-957) blocked production of IFN-γ and IL-2 by T cells in mouse models of arthritis, thereby reversing disease symptoms along with reducing cellular infiltration in arthritic lesions and autoantibody levels. Further investigations into the role of immunoproteasomes in mouse T cells showed that immunoproteasome deficiency or blocking of PSMB8 (β5i) function impaired Th1 differentiation without affecting Th2 differentiation. Moreover, the equilibrium between Th17 and Tregs shifted toward the latter, most likely by inhibiting the phosphorylation of STAT3 [38]. Altogether, both direct and indirect effects of PIs on suppression of T cell activation seem beneficial from an RA therapeutic perspective [1].

### Dendritic cells

DCs are the most powerful professional antigen-presenting cells and also control other players and processes of the immune system through the release of regulatory or stimulatory cytokines, depending on their developmental stage and activation state. DCs, for instance, control peripheral tolerance by inducing either anergy or depletion of autoreactive T cells. Two major DC subsets are derived from a common hematopoietic progenitor cell and abnormalities in both myeloid DCs as plasmacytoid DCs are associated with the development, maintenance and progression of several autoimmune diseases [39]. For their development and function, DCs heavily rely on the UPS and thus the effects of proteasome inhibition on the functionality of DCs have been the subject of extensive studies. In myeloid DCs, proteasome inhibition by BTZ reduced the expression of the DC-specific subset and activation markers (CD40, CD86, CD80, HLA-DR, CD206, CD209 and CD83) and induced apoptosis (upregulation of the pro-apoptotic protein Bax), inhibition of Toll-like receptor signaling, and suppression of cytokine release (IL-12, TNFα) due to impaired nuclear translocation of the NF-κB subunits RelA and RelB. Proteasome-inhibited DCs also failed to prime allogeneic T cells [6,29]. This susceptibility depended on the DC maturation stage as immature DCs appeared to be much more prone to respond to BTZ than mature DCs [40]. In other pre-clinical models of plasmacytoid DCs, effects of proteasome inhibition with BTZ, and also carfilzomib (CFZ) and ONX 0914, established inhibition of Toll-like receptor trafficking, IFN-α and IL-6 production and induction of apoptosis [41,42]. DC development and function upon differentiation in the presence of IFN-α were as equally sensitive to inhibition by BTZ as by the immunoproteasome inhibitor ONX 0914 (Verbrugge and colleagues, submitted).

Importantly, all these preclinical evaluations of PI-induced effects on human DCs span a relatively short drug exposure time and involve models of inflammatory DC differentiation, whereas clinical efficacy in chronic inflammation is often manifested after multiple cycles of drug administration and will also impact steady-state DC development [23]. Examining the impact of long-term BTZ exposure in CD34^+^ DC progenitors in a sustainable cell line model of steady-state Langerhans cell differentiation revealed early differentiation in precursor cells along with enhanced cytokine-driven Langerhans cell differentiation and maturation. Mechanistically, this effect was associated with enhanced NF-κB subunit RelB activation (Verbrugge and colleagues, submitted). Blanco and colleagues [39] reviewed their cytokine-induced monocyte-to-DC activation model that was created to demonstrate the contribution of inflammatory DCs arising under the influence of TNFα and type I IFNs (IFN-α and -β) to the development of autoimmunity. In particular, a pathogenic excess of IFNα/β in SLE, SS, dermatomyositis and early stages of psoriasis, along with an excess of TNFα in RA, inflammatory bowel disease, Crohn’s disease and psoriasis, may induce the generation of detrimental monocyte-derived inflammatory DCs. Targeting the production of both TNFα by myeloid DCs and type I IFNs by plasmacytoid DCs in these diseases might therefore be an attractive strategy to dampen chronic inflammation while maintaining protective immunity. This concept proved valid in an IFNα-driven monocyte-to-DC differentiation model exposed to BTZ and ONX 0914 (Verbrugge and colleagues, submitted).

### B lymphocytes

Depending on their developmental phase, B cells are involved in antigen presentation to T cells, the regulation of immune responses through cytokine production or the production of antibodies. Over the past decade, B-cell-depleting agents such as the CD20-targeted antibody rituximab have highlighted the essential role B cells play in the pathogenesis of various autoimmune diseases, including RA and SLE [43]. The UPS is critically involved in B-cell development and function, for example through its regulation of CD20 expression, the expression of the B-cell receptor and B-cell antigen presentation [44]. Van Anken and colleagues [45] reported a series of changes occurring during differentiation of B cells into plasma cells, starting with the expansion of metabolic capacity and the secretory machinery to accommodate the massive IgM production by plasma cells. Initially, it was rationalized that the increased protein turn-over in these cells would be accompanied by increased proteasome activity and that this feature would render them particularly susceptible to proteasome inhibition and accumulation of polyubiquitinated proteins, eliciting an apoptotic response. Later studies indicated that this increased susceptibility to proteasome inhibition was not necessarily due to increased proteasome levels and activity, but rather to stabilization of pro-apoptotic proteins leading to cell death [46]. Notably, several rodent models exhibited this increased susceptibility of plasma cells to proteasome inhibition. Neubert and colleagues [26] showed that reduced nephritis in mice with lupus-like disease was attributed to BTZ-induced plasma cell reduction. Beneficial effects from BTZ therapy were observed also in an experimental model of myasthenia gravis [47]. Also, selective inhibition of the immunoproteasome by ONX 0914 suppressed progression of experimental arthritis accompanied by a decrease in circulating levels of autoantibodies [27]. The next-generation PIs delanzomib and CFZ also showed promising anti-inflammatory effects by reducing autoantibody levels in mice with lupus [42]. These preclinical findings hold promise for the treatment not only of autoimmune diseases with increased plasma cell levels, but also for those in which autoantibody levels are critical determinants. Lastly, *in vitro* studies with human B lymphoblastoid cells showed that chronic exposure to BTZ led to upregulated CD20 expression levels due to impaired proteasome-mediated CD20 degradation. This feature may set a rationale for combination treatment of PIs with anti-CD20/rituximab [48].

## Mechanisms of proteasome inhibitor resistance and implications for autoimmunity

Regardless whether it concerns classical DMARDs, biological agents or experimental drugs, loss of efficacy due to primary or acquired resistance remains a recurrent theme in sustaining long-term therapeutic effects during treatment of autoimmune diseases [49,50]. For the PI BTZ, emergence of acquired resistance has been associated with reduced efficacy in the treatment of hematological malignancies [23]. In an autoimmune disease setting, where patients often require chronic drug administration, long-term efficacy may also be potentially hampered by the emergence of resistance phenomena. Over the past years, studies based on *in vitro* model systems revealed several mechanisms that may contribute to diminished efficacy with PIs and acquisition of resistance. One mechanism of PI resistance relates to the increased expression of proteasome subunits as a primary response to overcome PI targeting. In this respect, PSMB5 (β5) subunits were often found upregulated upon BTZ exposure in BTZ-resistant cells to compensate for inhibitory effects on proteasome activity [51]. Interestingly, Martinez-Gamboa and colleagues [52] observed that intrinsic factors and aberrant proteasome activation established dysregulation of β1i proteasome subunit expression in B lymphocytes of SS patients, conferring diminished PI inhibition and resistance to apoptosis. PI exposure and resistance may also provoke alterations in the relative composition of constitutive and immunoproteasome subunits, the impact of which on immune functions is not clear [53,54]. Another mechanism of acquired resistance to PIs is mediated by point mutations in the *PSMB5* gene encoding the PSMB5 (β5) subunit. This introduces amino acid alterations in the BTZ binding pocket of the PSMB5 (β5) subunit of the proteasome and confers diminished efficiency of BTZ binding. These mutations have been described in BTZ-resistant hematological cell line models, but have not yet been reported in biopsies or cell samples from patients experiencing clinical resistance to BTZ [51,53,55]. Interestingly, nature provides a precedent for *PSMB5* mutations as the marine bacterium *Salinospora tropica* generates self-resistance to the PI salinosporamide A (marizomib) it produces by upregulating a mutated form of the PSMB5 (β5) subunit that harbors a single amino acid substitution identical to those found in hematological cell lines with acquired BTZ resistance [51,53]. A third recognized resistance mechanism operates via selective drug efflux transporters of the ATP-binding cassette family, known to confer multidrug resistance, which could facilitate cellular extrusion of specific PIs [56]. Whereas BTZ appeared to be a relatively poor substrate for drug efflux transporters, next-generation PIs such as CFZ, ONX 0912 (oprozomib) and ONX 0914 were found to be proficient substrates for the efflux transporter MDR1 (P-glycoprotein (Pgp)). Of interest, inhibition of Pgp could reverse resistance to these [51,54]. Since Pgp is constitutively expressed on lymphocytes from RA patients, and its expression is correlated with disease activity, both basal levels of expression and upregulated levels upon long-term administration could be limiting factors in sustaining long-term efficacy [56].

Primary resistance to BTZ has been associated with upregulated expression of heat shock proteins [57]. This could be of relevance for PI treatment of autoimmune diseases since enhanced expression of heat shock proteins has been described in various inflammatory conditions, such as RA, type 1 diabetes, and atherosclerosis. Finally, impaired inhibition of NF-κB and activation of PI3K/Akt pro-survival pathways through increased secretion of the insulin-like growth factor (IGF)-1 and enhanced activation of the IGF-1 receptor may also contribute to BTZ resistance [58]. This latter notion could have implications for the treatment of autoimmune diseases since IGF-1 is produced by plasma cells, in the bone marrow microenvironment, and under various disease conditions. Together, the expanded knowledge of molecular mechanisms that could impact (pre)clinical activity of various PI subclasses may be exploited for rational clinical application and to support optimal efficacy.

## Current clinical status of proteasome inhibitors in autoimmune disease

Other than for malignant diseases and in the setting of graft-versus-host disease, clinical application of BTZ or next-generation PIs in the treatment of autoimmune diseases is still at an early stage. Moran and colleagues [6] discussed a few case reports describing clinical experience with BTZ in immune-mediated diseases. These include reports of patients with autoimmune diseases such as SLE and refractory autoimmune hemolytic anemia (chronic cold agglutinin disease) where beneficial effects of BTZ were accompanied by improved laboratory findings consisting of reduced anti-extractable nuclear antigen levels, reduced anti-double-stranded DNA antibody levels, and normalization of complement levels and platelet count. These studies were further corroborated by Hiepe and colleagues [59], who reported that refractory SLE patients responded to BTZ therapy. Currently, there are only three registered clinical trials for BTZ in autoimmune diseases and these include refractory cold agglutinin disease, IgA nephropathy and proliferative lupus nephritis, the last of which has been withdrawn prior to initiation [60]. Registered clinical trials for autoimmune diseases with next-generation PIs (for example, CFZ, oprozomib, delanzomib or ONX 0914) are still lacking.

Obviously, successful implementation of PI therapies in autoimmune diseases will be dependent on managing toxic side effects. In early clinical studies of BTZ treatment of multiple myeloma, peripheral neuropathy emerged as one undesirable side effect, though others were also documented - for example, pancytopenia, congestive heart failure, pulmonary edema, gastrointestinal hemorrhage, disseminated herpes zoster, sepsis and renal failure [23]. Peripheral neuropathy appeared to be related to non-proteasomal inhibition by BTZ of HtrA2/Omi, a neuronal survival protease [61]. Currently, modified schedules and routes of administration (subcutaneous versus intravenous) and (orally) available second generation PIs have improved their efficacy, safety and toxicity profiles [23].

## Future perspectives/conclusion

There is still a long way to go before the transition of PIs from experimental to standard therapeutics. For this to occur in RA treatment PIs would have to be better than the powerful and relatively safe anti-rheumatic drugs that are already on the market or they should work better in combination with conventional DMARDs and/or biologics. However, for autoimmune diseases other than RA for which therapeutic options are more limited - for example, SLE, SS and scleroderma - PIs deserve further consideration.

In conclusion, preclinical evaluation of BTZ and next-generation PIs for the treatment of autoimmune diseases such as RA is generally promising. PIs have the capacity not only to induce cell-specific apoptosis (for example, of autoantibody-producing plasma cells), but also to target pro-inflammatory cytokines and their receptors and disrupt intracellular signaling pathways in pro-inflammatory immune effector cells.

Lessons learned from preclinical and clinical observations with BTZ in hematological malignancies indicated that the timing and scheduling of drug administration is a key factor in optimized treatment and to minimize BTZ-related toxicity, side effects from which include peripheral neuropathy, thrombocytopenia, diarrhea and an increased risk of developing infectious complications [6,23]. These observations represent clinically relevant factors to consider in the treatment of autoimmune/inflammatory disorders, in particular when drug administration would be more chronic. The risk of thrombocytopenia and polyneuropathy might be more acceptable in patients with malignant diseases than in those suffering from chronic autoimmune diseases. Notably, reduction of side effects accompanied by similar levels of efficacy were observed upon subcutaneous BTZ administration [23]. Beyond this, the next generation PI CFZ and the orally delivered next-generation PIs MLN9708 or ONX 0912 (oprozomib) might also overcome these toxicities.

Experience from PI treatment of hematological malignancies will expand insights into mechanisms related to toxicity or the development of resistance. Meanwhile, the role of the immunoproteasome and the possible advantages of its specific targeting merit further investigation and might prove to be key in the reduction of chronic inflammation with acceptable unwanted side effects.

## Abbreviations

BTZ: Bortezomib
CFZ: Carfilzomib
DC: Dendritic cell
DMARD: Disease-modifying anti-rheumatic drug
IFN: Interferon
IGF: Insulin-like growth factor
IL: Interleukin
MHC: Major histocompatibility complex
NF: Nuclear factor
Pgp: P-glycoprotein
PI: Proteasome inhibitor
RA: Rheumatoid arthritis
SLE: Systemic lupus erythematosus
SS: Sjögren’s syndrome
TNF: Tumor necrosis factor
Treg: regulatory T cell
UPS: Ubiquitin-proteasome system
